# Everybody's Page

**Published:** 1890-01-25

**Authors:** 


### Everybody's Page.

Health depends more on precautions than on remedies.

Ripe tomatoes will remove ink stains, not only from the hands, but from white cloth.

In China eight varieties of leprosy are recognised, and the disease is considered contagious, infectious, and hereditary, but is said to disappear in four generations. There are leper villages to which all suffering from the disease are sent.

An old-fashioned remedy for relieving ear-ache is to put a good-sized fig into the oven and get it quite hot, then wrap it up in a thin handkerchief and tie it on over the affected ear; it retains the heat for a long time, and is very soothing.

The contrast between wealth and poverty was clearly defined in the mind of the native Indian who wrote that the rich man wallows on velvet cushions, while the poor man snorts on flint. Certainly flint is more calculated to make a man snort than to allow him to wallow.

When pasting labels on to iron, tin, zinc, or any other metal surface with common paste, glue, or gum-arabic, it will be found advantageous, in order to make the labels adhere thoroughly, first to rub onion juice over the place of contact. It adheres in a most decisive manner to any metal on which it has been rubbed.

In America several bacteriologists have pronounced sulphur fumes to be quite useless for disinfection. The *Medical Record* says that Dr. J. G. Johnson states that he has propagated diphtheria from pieces of blankets which had been thoroughly fumigated by sulphur, and in New York and Brooklyn currents of steam are being advised for disinfecting purposes.

Loofahs, which so many people use now as flesh rubbers in their baths, are the fruit of a sort of climbing vine indigenous to Egypt and Arabia, and also grown in the West Indies and some places in Africa. When being prepared for washing purposes, the green epidermis is removed, and then the loofah is washed and beaten in water to remove all the pulp and seeds, leaving nothing but the light network of the fruit.

Electric light is very lovely and very useful, and in private houses it is especially fascinating to just touch a little button and turn it on in a second without any trouble, but it is very sad to hear from New York of several accidents occurring in the last few months, due, it would seem, to the difficulties to contend with in the distribution of electricity. Let us hope we shall be more fortunate in London. When wires are underground they shock pedestrians sometimes, and an electric shock, unasked and unexpected, however slight, must be anything but pleasant.

An old Hungarian lately died who refused medical aid as he believed only in "healing by faith." There is a class of people belonging to a place in London called Bethshan who in time of sickness will have no doctor, as they say they have an implicit belief in God's power and willingness to heal every sort of disease, and that is enough for them. The cures said to have been effected amongst the believers in faith-healing amount to being miraculous; cripples who have used crutches for years suddenly get up and walk away, and every sort of disease is instantaneously cured. The old Hungarian we mention died of chronic bronchitis. It is a hard faith which teaches men to reject the services of medical science, perhaps the greatest blessing that has been given us.

Snake's liver tastes very like game, in fact, like good ptarmigan. Certainly there is plenty of food in the world if people like Mr. Frank Buckland do not mind experimentalising on new diet.

It appears from the discussions at the Hygienic Congress at the recent Paris Exhibition that profits are made out of the removal of refuse from houses in France, for the removal of which there are not paid contractors, as in this country.

The dust we see in a sunbeam is, according to Dr. Marcet, F.R.S., of an organic nature, consisting of countless motes. Dust when mixed with air is inflammable, and in flour mills and coal mines has frequently been the cause of explosions.

Not long ago in Vienna a thief snatched a bag from the son of a hospital porter who was walking along in the street. Picture the feelings of the thief when, on opening the bag, he found it contained nothing but a human head intended for examination.

A great many of the handles of our dinner knives are made of celluloid. Manufacturers say that it keeps its colour much better than ivory, and will stand washing in hot water, and unless brought into immediate contact with fire, no amount of heat will cause combustion.

The Young Women's Christian Association have opened two gymnasiums, one at 14, Finsbury-square, and one at 316, Regent-street, and at twenty-two institutes they teach shorthand, typewriting, arithmetic, bookkeeping, dress cutting, cookery, ambulance nursing, and many other useful things. Examinations are held on these subjects, and prizes and certificates given.

Few people will be tempted to quit this world by means of charcoal fumes if they read the dying impressions of two Frenchmen who asphyxiated themselves in that manner, one thirty years ago and one recently, and they both wrote down their feelings for the benefit of mankind. The first effect of charcoal is to give an intense headache, and the agony seems to increase, for one man wrote "it is too much to suffer," and the other "I did not think it would be such agony to die."

When influenza was raging in 1851, Mr. Richard Doyle wrote a letter to Lady Duff Gordon (published some months ago), showing that the depressed state of people's feelings was pretty much the same then as it is now. He writes that at a party at Lady Molesworth's every lady was sneezing and blowing her nose, every gentleman sighing and feeling feverish and "seedy," and there was a low murmur on the surface of "society" of "Let's go home." The conversation of the night was not brilliant, consisting chiefly of the words "Have you got it yet?"

What's in a name? A very great deal, so some of the tradesmen in London think, and rightly too. A bootmaker who drops the plain John Brown or Jones, as the case may be, and puts up in large letters over his shop "Phit-eesi," is to be commended for his accurate hit at the quality which the weary pedestrian most wishes his boots to possess. Then there is the "So-al" machine; think what that name suggests to many of the female passers-by, who instantly conjure up the wildest dreams of this wonderful machine's capacity for work, and go in and buy forthwith. Also in our daily peregrinations we notice "The Tit-Bits Refreshment Bar." Now we think that most likely, if we had to get our dinner at a cooked meat shop, this would appeal to our feelings very much indeed, and the wily shopman thinks so too.

